# Determinants of adverse management outcomes of blunt abdominal trauma patients operated at a referral hospital in southern Ethiopia: a retrospective record review

**DOI:** 10.1186/s12893-023-02261-7

**Published:** 2023-11-21

**Authors:** Demoz Abraha, Essay Gebreyes, Eskinder Wolka, Getahun Dender, Abebe Sorsa, Joshua Muhumuza

**Affiliations:** 1 Department of Surgery, College of Heath Sciences and Medicine, Wolaita Sodo University Teaching and Referral Hospital, Wolaita Sodo, Ethiopia; 2 Department of surgery, Faculty of clinical medicine and dentistry, Kampala international university- western campus, Ishaka-Bushenyi, Uganda

**Keywords:** Adverse management outcomes, Blunt abdominal trauma, Southern Ethiopia

## Abstract

**Background:**

Abdominal trauma is one of the common reasons for emergency visits yet there is paucity of data about the subject in the horn of Africa. This study was aimed at determining the determinants of adverse management outcomes of blunt abdominal trauma among operated patients at Wolaita Sodo University Teaching and Referral Hospital, Ethiopia.

**Methods:**

This was a three-year retrospective review conducted among 128 patient records selected using purposive sampling in which all records for the patients operated for a diagnosis of blunt abdominal trauma during the study period were included. A pretested checklist was used to extract the data relating to adverse outcomes and characteristics of the patients. A descriptive analysis followed by logistic regression was done.

**Results:**

Of the 128 patients, adverse management outcomes related to blunt abdominal trauma occurred in 52%. Patients residing in rural areas (adjusted odds ratio 3.23, 95% confidence interval: 1.13–9.24) and those with tachycardia, (adjusted odds ratio = 3.25, 95% confidence interval: 1.19–8.83) or tachypnea (adjusted odds ratio 3.25, 95% confidence interval: 1.19–8.83) were more likely to have adverse management outcomes.

**Conclusion:**

Adverse management outcomes are relatively high and associated with rural residence and deranged vital signs (tachycardia and tachypnea). Close monitoring targeting patients from rural residence and those presenting with tachycardia and tachypnea is recommended.

## Introduction

Trauma is the commonest cause of hospitalization. It remains the most common cause of death and disability for all individuals between the ages of 1 and 44 years and is the third most common cause of death regardless of age. In 2004 there were 29.6 million injured patients treated in emergency departments, out of whom approximately 167,000 died [1]. In that year Ethiopia ranked 3rd for trauma related deaths in Africa following Nigeria and South Africa. World Health Organization (WHO) global burden of injury estimated that 5 million injury related deaths occur annually with the highest injury-related mortality rates among men in Africa. The mortality is high in the low- and middle-income countries but lower in the industrialized world due to better prevention and therapeutic modalities. Africa has the highest trauma related mortality due to poor infrastructure and traffic control plus inadequate Emergency management as compared to other continents [2]. Unintentional injuries are responsible for over 110,000 deaths per year. Motor vehicle collisions account for over 40%. It is also the leading cause of years of productive life lost. Injury-related medical expenditures are estimated to be $117 billion each year in the United States. The aggregate lifetime cost for all injured patients is estimated to be in excess of $260 trillion [1].

The abdomen is the third commonly injured region, and 40 to 80% of deaths after trauma are due to exsanguination caused by injuries to the abdominal organs [2]. A study in Ethiopia revealed that abdominal trauma accounts for about half of all surgical emergency visits [2]. From a prospective study at Menilik II hospital in Ethiopia, 90% of the trauma related deaths occurred in the first 24 h, 5% in the second day and 2.3% after day seven [3]. In most literatures, blunt abdominal trauma is more common (80–90%) than penetrating [4], but there are some institution-based studies from Nigeria and Kenya which showed penetrating abdominal injury to be more common. The spleen is the most commonly injured solid organ in blunt trauma while hollow organs are most injured in penetrating [2]. One quarter of abdominal trauma requires emergency laparotomy. Currently non operative management (NOM) reaches around 90% especially in children. Despite being one of the common emergencies requiring laparotomy, there is paucity of data relating to outcomes of blunt abdominal trauma surgery in the horn of Africa. This study determined the determinants of management outcome of blunt abdominal trauma among operated patients at Wolaita Sodo University Teaching and Referral Hospital (WSUTRH), Ethiopia.

## Methods

### Study area

The study was conducted at Wolaita Sodo University Teaching & Referral Hospital (WSUTRH), located in Wolaita Sodo town, Ethiopia. It’s one of the biggest hospitals in Wolaita zone serving as a referral center for patients not only from Wolaita zone but also for all the surrounding zones. The Hospital has diversified departments among which is the Surgical department. Surgery department has thirteen surgeons among whom seven are general surgeons, the others being orthopedic, maxillofacial and Uro-surgeons. The department has fourteen nurses and fourteen anesthetists shared with gynecology and obstetrics department. The annual admission rate is 1435. There are 63 beds in surgical ward and 11 beds in adult emergency room. The surgical department has one operation room in which there are two major operation tables with adequate equipment. There is one ICU center in the hospital with four beds shared among all departments.

### Study design and period

This was a retrospective record review conducted from November 1, 2017 up to October 31, 2020 (a period of three years). This period was chosen because it was the maximum period for which the patient records could be accessed.

### Study population

All patients operated at Wolaita Sodo University Teaching & Referral Hospital (WSUTRH), for a diagnosis of blunt abdominal trauma (BAT) during the study period with available medical records were considered for this study.

### Eligibility criteria

The inclusion criteria was: all patients operated for a diagnosis of BAT with available medical record including both adults and children. The exclusion criteria was: patients with a medical record that had insufficient documentation.

### Sample size determination

Since we aimed at attaining the maximum possible power, all the records that could be accessed were reviewed. Since the records that could be accessed were records for the patients managed over the previous 3 years, all the records for the patients managed between the period of November, 2017 and October 2020 were reviewed irrespective of the number.

### Sampling procedure/technique

Purposive sampling was done in which all records for the patients operated at WSUTRH for a diagnosis of blunt abdominal trauma (BAT) during the study period were sampled.

### Data collection methods

Data were collected from patient’s medical record cards and operation registry by using a pre-tested data collection sheet (data extraction check list). Residents, interns and staff from card room were involved in the data collection processes. They were trained on how to collect the relevant data using the data collection sheet. The principal investigator was continuously supervising the data collectors. Patient documents were reviewed using checklist.

### Study variables

The primary outcome of interest was unfavourable management outcome which was the dependent variable. In this study, unfavourable management outcome referred to atleast one of the following: anemia, surgical site infection, respiratory complications, hemorrhage, ICU admission or death. We hypothesized that different socio demographic, injury related, and clinical factors could predict the outcome of BAT operative management and these constituted the independent variables. These included: Socio demographic (age, sex), mechanism of injury (MVA and car, or other), presenting symptom, vital signs at presentation, duration from trauma to arrival for operation, organ injured, intervention at emergency, type of operation, comorbidity and grade of organ injury. The data relating to all these variables was collected using the checklist.

### Operational definitions

Surgical site infections: infection following surgical incisions which includes all types documented in the card by the attending physician before patient discharge. Anemia: hemoglobin level below 7 mg/dl for whom blood was transfused.

### Data quality control

The data collecting sheet was standardized by testing it on 12 patient records before the study to make sure that the data collecting sheet was capable of yielding the required data for the study and modifications were made according to the results. The study assistants were trained on how to collect the relevant data using the data collection sheet. The principal investigator was continuously supervising the data collectors.

### Methods of data analysis

Data was entered into Microsoft Excel 20.0 and imported into Stata version 15.0 for analysis. Descriptive statistics was done to determine the proportions of socio-demographic characteristics and prevalence of unfavourable management outcome. Chi-square analysis was performed to determine the association between factors and management outcome. Bivariable followed by multivariable logistic regression were performed. Odds ratios, 95% Confidence Intervals and *p* values were presented. Independent variables with p < 0.05 were considered for multivariate model and backward regression done. A factor was considered a significant determinant of adverse management outcome if its p-value was < 0.05.

## Results

### Characteristics of respondents

A total of 128 subjects were included in this study, 79.7% were males and 81.3% lived in rural places. Participants were 1 to 70 years old with a mean age of 26 ± 15 years and the majorities were adults (78.1%). Only 20.3% were referred from other health center or hospital. A total of 55.5% were related to other types of accidents such as falls, animal kick and fighting injuries. Only 4.7% of the participants had some other comorbidity and 17.2% had trauma other than abdomen. Of the 128 patients, 72% had tachycardia, 80.5% had tachypnea at arrival. At emergency 98.4% of them were resuscitated. The rest of population characteristics are shown in Table 1 below.

**Table 1:** Baseline population characteristics

Population Characteristics	Categories	Frequency	Percent
Sex	Male	102	79.7
	Female	26	20.3
Age (years) Mean age 26 ± 15 years	< 18 years	28	21.9
	≥ 18 years	100	78.1
Residence	Rural	104	81.3
	Urban	24	18.8
Means of arrival	Referred	26	20.3
	Self-referred	102	79.7
Mechanism	MVA and car accident	57	45.5
	Other	71	55.5
Trauma to other regions	Yes	22	17.2
	No	106	82.8
Comorbid illness	Yes	6	4.7
	No	122	95.3
Surgery done > 24 h from admission time	Yes	29	22.7
	No	99	77.3
Intra operative finding	Hemoperitoneum	80	62.5
	GIT injury	47	36.7
**Vital sign at arrival**			
Low Blood Pressure	Yes	61	47.7
	No	67	52.3
Tachycardia	Yes	92	71.9
	No	36	28.1
Tachypnea	Yes	103	80.5
	No	25	19.5
Febrile	Yes	41	32.0
	No	87	68.0
**Interventions**			
Fluid resuscitation	Yes	126	98.4
	No	2	1.6
Blood transfusion	Yes	9	7.0
	No	119	93.0
Antibiotics initiation	Yes	65	50.8
	No	63	49.2
**Organs Injured**			
Solid organs	liver	2	1.6
	spleen	74	57.8
	kidney	1	0.8
Hallow organs	stomach	1	0.8
	Jejunum	19	14.8
	ileum	26	20.3
	colon	1	0.8
	other	5	3.9
**Procedures performed**			
Solid organ	liver repair	2	1.6
	splenectomy	58	45.3
	spleen salvaging procedures	15	11.7
	Left Nephrectomy	1	0.8
Hallow organs	bowel repair	36	28.1
	bowel resection and anastomosis	9	7.0
	stoma	1	0.8
	nontherapeutic laparotomy	4	3.1
	other	3	2.3

### Determinants of adverse outcomes

In the final model, accounting for the other factors three factors were found to be significantly associated with unfavourable management outcome (complications related to BAT) at p-value < 0.05. Compared to those who reside in urban settings, those who live in rural settings were more likely to experience complications related to BAT (adjusted odds ratio aOR 3.23, 95% Confidence Interval (CI):1.13–9.24). Patients who had tachypnea or tachypnea were about three times more likely to experience complications related to BAT (aOR 3.25, 95% CI:1.19–8.83) (Table 4). Tables 2 and 3 show the outcomes and bivariable analysis respectively.

**Table 2:** Management outcomes

Outcome	Categories	Frequency	Percent
Complications (Anemia, surgical site infection, respiratory complications and hemorrhage)	Yes	66	51.6
	No	62	48.4
ICU admission	Yes	12	9.4
	No	116	90.6
Death	No	121	94.5
	Yes	7	5.5

**Table 3:** Bivariable analysis for determinants of adverse management outcomes of blunt abdominal trauma patients

Determinants		Adverse Management Outcomes (N = 128)		COR (95%CI)		P
		Yes (n = 66)	No (n = 62)			
Sex	Male	53(52.0)	49(48.0)	1		
	Female	13(50.0)	13(50.0)	1.08(0.46–2.56)		0.858
Age (years)	< 18 years	18(64.30	10(35.7)	1		
	≥ 18 years	48(53.3)	42(46.7)	1.58(0.66–3.79)	0.310	
Residence	Rural	59(56.7)	45(43.3)	1		
	Urban	7(29.2)	17(70.8)	13.18(1.22–8.33)		0.018*
Means of arrival	Referred	10(38.5)	16(61.5)	1		
	Self-referred	56(54.9)	46(45.1)	0.66(0.20–2.15)		0.4893
Mechanism	RTA	29(50.9)	28(49.1)	1		
	Other	37(52.1)	34(47.9)	0.95(0.47–1.91)		0.889
Trauma to other than abdomen	Yes	11(50.0)	11(50.0)	1		
	No	55(51.9)	51(48.1)	0.93(0.37–2.32)		0.872
Comorbid illness	Yes	6(100.0)	0(0.0)			0.079
	No	60(49.2)	62(50.8)	-		
Low Blood Pressure	Yes	35(57.4)	26(42.6)	1		
	No	31(46.3)	36(53.7)	1.56(0.78–3.14)		0.210
Tachycardia	Yes	53(57.6)	39(42.6)	1		
	No	13(36.1)	23(63.9)	2.40(1.08–5.33)		0.031*
Tachypnea	Yes	58(56.3)	45(43.7)	1		
	No	8(32.0)	17(68.0)	2.74(1.08–6.92)		0.033*
Febrile	Yes	19(46.3)	22(53.7)	1		
	No	47(54.0)	40(46.0)	0.74(0.35–1.55)		0.417
Fluid resuscitation	Yes	66(52.4)	60(47.6)			0.275
	No	0(0.0)	2(100.0)	-		
Blood transfusion	Yes	4(44.4)	5(55.6)	1		
	No	64(53.8)	55(46.2)	0.69(0.18–2.69)		0.590
Antibiotics incitation	Yes	27(41.5)	38(58.5)	1		
	No	39(61.9)	24(38.1)	0.44(0.22–0.89)		0.022
Surgery done > 24 h from admission time	Yes	17(58.6)	12(41.4)	1		
	No	49(49.5)	50(50.5)	1.45(0.63–3.34)		0.388
Grade	Grade I and II	20(41.7)	28(58.3)	1		
	Grade II, IV and V	43(57.3)	32(42.7)	0.53(0.26–1.11)		0.09

*p < 0.05. N/A = Logistic regression not done since there was no outcome of interest in one of the categories

**Table 4:** Multivariable analysis for determinants of adverse management outcomes of blunt abdominal trauma patients

Determinants	Categories	Adverse outcomes	p-value
Residence	Rural	3.23 (1.13–9.24)	0.047
	Urban	1	
Tachycardia	Yes	3.25 (1.19–8.83)	0.034
	No	1	
Tachypnea	Yes	3.25 (1.19–8.83)	0.029
	No	1	

## Discussion

This study aimed to identify the determinants of adverse outcomes following laparotomy for blunt abdominal trauma (BAT). In our study, 52% of patients had post-operative complications, and 6% died, whereas in the study conducted in St Paul’s Hospital Millennium Medical College (SPHMMC), 17.8% of the patients had post-operative complications lower than our study, though the mortality rate was slightly higher (8.5%) but comparable to that seen in our study [2]. The difference seen in the proportion of the complications could be arising from the difference in the study populations given that our study only included blunt abdominal trauma yet the study at SPHMMC had majority of the participants with penetrating trauma (62%). In the SPHMMC study, it was noted that complications were more in the patient with blunt abdominal trauma compared to those with penetrating abdominal trauma. This could be because in penetrating abdominal trauma, the threshold for laparotomy is low in which even patients with no injured organs may undergo laparotomy yet with blunt abdominal trauma, laparotomy is only done when there is evidence of intra-abdominal organ injury requiring surgical intervention.

In another study conducted in Tanzania, 42% of the patients had post-operative complications which was not much different from the percentage seen in our study but the mortality rate was 13.2% which was about twice the mortality seen in our study [5]. The higher mortality in the Tanzania study compared to that seen in this study could be as a result of delayed intervention given that majority of the participants in the Tanzania study had delayed intervention compared to our study where only 22.7% were operated after 24 h. This time to operation was found to be significantly associated to mortality in the Tanzania study.

In another study conducted in India, mortality rate among cases of BAT was 4% [6]. In this study non therapeutic laparotomy rate was 3.1% slightly lesser to other studies in Addis Ababa (4.6%) and Tanzania (6.6%) [2, 5]. The lower mortalities noted in these studies are because these studies included both the patients that underwent operative management and those who were managed nonoperatively.

In our study the most affected organ was spleen accounting for 58% of all BAT followed by ileum. This was similar to other studies in Addis Ababa, Tanzania, Qatar and India where the most commonly injured organ in BAT was the spleen [2, 6–9]. Also in our study, 45% of the BAT was due to motor vehicle accidents comparable to the findings in Tanzania (53.7% RTA), Egypt (62.8%), India (53%) and Qatar (61%) [6–9]. The reason for road traffic accidents causing a lot of blunt abdominal trauma is possibly because a big number of motorists do not follow the traffic regulations as reported by Olasinde et al. [10] which increases the risk for road traffic accidents resulting in blunt abdominal trauma due to the high energy of impact.

Patients residing in rural areas were at increased odds of getting complications related to BAT which was in agreement with the findings in Canada [11], Australia [12] and Merryland [13]. This may be related to the diverse socio-demographic and health system related factors associated with people living in rural areas. People living in rural areas are often low income and may have limited health literacy contributing to delayed health care. Moreover, people living in rural areas may also have problems related to transportation which may subsequently result in complications related to delayed care.

We also noted that patients who had tachycardia or tachypnea were more likely to have adverse outcomes. This may be because deranged vital signs are associated with hemodynamic instability which hemodynamic instability has been reported to be associated with a dverse outcomes among trauma patients in a number of studies [14–18].

### Limitations

This study had some limitations. First, record review has its own limitation in that not all factors related to the outcome are recorded. Some of the study variables may not be found in the chart and some charts of patients were lost from card room. Second, our categorization of rural and urban was based on how the patient defined their residence.

## Conclusion

Adverse management outcomes related to BAT were common among patients in south Ethiopia. Rural residence, deranged vital sign (tachycardia and tachypnea) at arrival were the factors that led to a higher likelihood of adverse management outcomes. Prevention strategies to reduce and control complications related to BAT should specifically target patients from rural settings and those who present with deranged vital signs, especially tachycardia and tachypnea. These strategies should include close monitoring given that deranged vital signs were associated with adverse outcomes.

## Data Availability

The data used to support the findings of this study are available from the corresponding author upon request.

## Abbreviations

aOR: Adjusted odds ratio
BAT: Blunt Abdominal Trauma
CI: Confidence Interval
COR: Crude odds ratio
NCS: Non Complex surgery
NOM: Non Operative Management
RTA: Road Traffic Accident
SPHMMC: St. Paul’s Hospital Millennium Medical College
WSUTRH: Wolaita Sodo University Teaching & Referral Hospital
